# Virtual reality and artificial intelligence for 3-dimensional planning of lung segmentectomies

**DOI:** 10.1016/j.xjtc.2021.03.016

**Published:** 2021-03-16

**Authors:** Amir H. Sadeghi, Alexander P.W. M. Maat, Yannick J.H. J. Taverne, Robin Cornelissen, Anne-Marie C. Dingemans, Ad J.J. C. Bogers, Edris A.F. Mahtab

**Affiliations:** a Department of Cardiothoracic Surgery, Thoraxcenter, Erasmus Medical Center Rotterdam, Rotterdam, The Netherlands; b Department of Pulmonary Medicine, Erasmus Medical Center Cancer Institute, Rotterdam, The Netherlands

**Keywords:** virtual reality, preoperative planning, segmentectomy, video-assisted thoracoscopic surgery, lung cancer, 2D, 2 dimensional, 3D, 3 dimensional, AI, artificial intelligence, CT, computed tomography, DICOM, digital imaging and communication in medicine, NSCLC, non–small cell lung cancer, S, segment, VATS, video assisted thoracoscopic surgery, VR, virtual reality

## Abstract

**Background:**

There has been an increasing trend toward pulmonary segmentectomies to treat early-stage lung cancer, small intrapulmonary metastases, and localized benign pathology. A complete preoperative understanding of pulmonary anatomy is essential for accurate surgical planning and case selection. Identifying intersegmental divisions is extremely difficult when performed on computed tomography. For the preoperative planning of segmentectomies, virtual reality (VR) and artificial intelligence could allow 3-dimensional visualization of the complex anatomy of pulmonary segmental divisions, vascular arborization, and bronchial anatomy. This technology can be applied by surgeons preoperatively to gain better insight into a patient's anatomy for planning segmentectomy.

**Methods:**

In this prospective observational pilot study, we aim to assess and demonstrate the technical feasibility and clinical applicability of the first dedicated artificial intelligence-based and immersive 3-dimensional-VR platform (PulmoVR; jointly developed and manufactured by Department of Cardiothoracic Surgery [Erasmus Medical Center, Rotterdam, The Netherlands], MedicalVR [Amsterdam, The Netherlands], EVOCS Medical Image Communication [Fysicon BV, Oss, The Netherlands], and Thirona [Nijmegen, The Netherlands]) for preoperative planning of video-assisted thoracoscopic segmentectomies.

**Results:**

A total of 10 eligible patients for segmentectomy were included in this study after referral through the institutional thoracic oncology multidisciplinary team. PulmoVR was successfully applied as a supplementary imaging tool to perform video-assisted thoracoscopic segmentectomies. In 40% of the cases, the surgical strategy was adjusted due to the 3-dimensional-VR–based evaluation of anatomy. This underlines the potential benefit of additional VR-guided planning of segmentectomy for both surgeon and patient.

**Conclusions:**

Our study demonstrates the successful development and clinical application of the first dedicated artificial intelligence and VR platform for the planning of pulmonary segmentectomy. This is the first study that shows an immersive virtual reality-based application for preoperative planning of segmentectomy to the best of our knowledge.

## Graphical abstract

**Figure:**
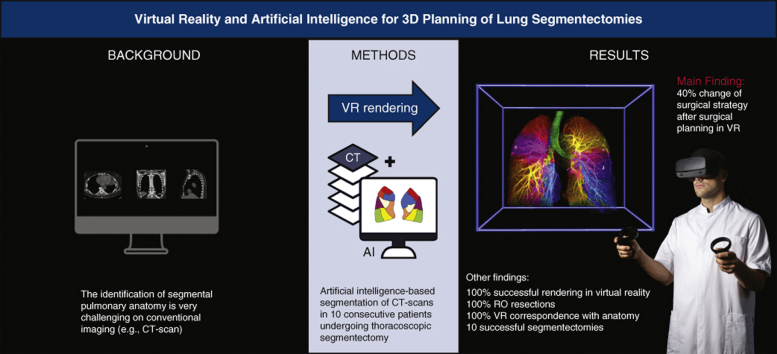

The identification of segmental anatomy for surgical planning of lung segmentectomy is quite challenging with conventional imaging. In the current study, a novel method is presented to create artificial intelligence based virtual reality reconstructions to prepare for thoracoscopic segmentectomies in 10 consecutive patients. *VR,* Virtual reality; *CT*, computed tomography.

**Figure:**
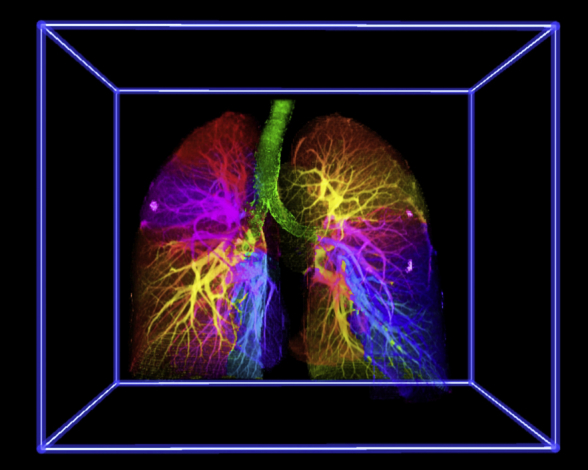
Artificial intelligence and virtual reality reconstruction of segmental pulmonary anatomy.

**Central Message**

PulmoVR, a novel and immersive virtual reality-based application, is a feasible and clinically applicable method for accurate and patient-tailored surgical planning of pulmonary segmentectomy.

**Perspective**

We present the development and application of an immersive virtual reality-based platform (PulmoVR) that enables personalized surgical planning for pulmonary segmentectomies. PulmoVR is the first dedicated virtual reality segmentectomy planning tool that provides accurate surgical planning for segmentectomy. Future clinical studies should demonstrate clinical benefits of such technology.

See Commentary on page 322.

Due to an increase in the frequency of small ground-glass opacity and incidental small intrapulmonary metastases detection, there has been an increasing trend toward lung-sparing resection techniques such as segmentectomy.^1^^,^^2^ In our hospital, we have adopted segmentectomy for specific surgical procedures, including resections of small (≤2 cm) stage I non–small cell lung cancer (NSCLC), benign intrapulmonary disease limited to segments (eg, bronchiectasis), and deeply located or nonpalpable metastases of other primary malignancies.

Thoracoscopic lung segmentectomies are generally more complex than thoracoscopic lobectomies.^1^ Identifying intraparenchymal planes and ligation of target branches of pulmonary arteries, veins, and bronchi can be more challenging, partly due to the existence of many anatomical variations and abnormalities in pulmonary vascular anatomy.^3^^,^^4^

Conventional computed tomography (CT) imaging is the gold standard for preoperative planning of anatomical (sub)lobar resections. Unfortunately, the identification of segmental borders and segmental branches of arteries, veins, and bronchi is challenging when conventional 2-dimensional (2D) CT is used.

The success of pulmonary segmentectomies largely depends on the surgeon's preoperative understanding of the anatomy. Previous studies have already demonstrated the feasibility and added value of 3-dimensional (3D) reconstructions of pulmonary anatomy for preoperative and intraoperative planning of pulmonary segment resections.^5^^,^^6^ Over the past few years, virtual reality (VR) devices and software are gaining popularity and have been shown to improve the understanding of patient anatomy by surgeons.^7^, ^8^, ^9^ A VR platform has more features and functions than existing 2D or 3D planning software, including immersive and interactive manipulation, realistic in-depth perception, and visualization of complex relationships of anatomic structures that are ready at hand and can be applied by surgeons to gain a more realistic insight into a patient's anatomy.^8^^,^^10^ Moreover, automated imaging algorithms could potentially create a more efficient planning by enabling automatic visualization of anatomic structures of interest.^11^^,^^12^ By developing an artificial intelligence (AI)-based and immersive 3D-VR platform as a supplementary preoperative planning tool to traditional CT imaging, a novelty can be added to the thoracic surgeon's armamentarium.

To overcome most of the shortcomings of the currently available imaging modalities and to create an easy-to-use and dedicated platform for CT imaging review, we have developed a 3D VR-based digital imaging and communication in medicine (DICOM) viewer. In addition, we developed PulmoVR (jointly developed and manufactured by Department of Cardiothoracic Surgery [Erasmus Medical Center], MedicalVR, EVOCS Medical Image Communication [Fysicon BV], and Thirona), a dedicated immersive 3D-VR and AI-based segmentectomy surgical planning tool. We hypothesized that such a platform could provide a more accurate preoperative understanding of the patient-specific segmental and intersegmental anatomy.

The present prospective single-center pilot study shows our early results on the clinical application of our innovative immersive AI and VR-based segmentectomy planning tool (PulmoVR) in 10 consecutive patients. The main objectives are to assess the technical feasibility and clinical applicability of PulmoVR in performing 3D VR-guided video-assisted thoracoscopic surgery (VATS) segmentectomies.

## Methods

### Patient Selection

**Figure 1:**
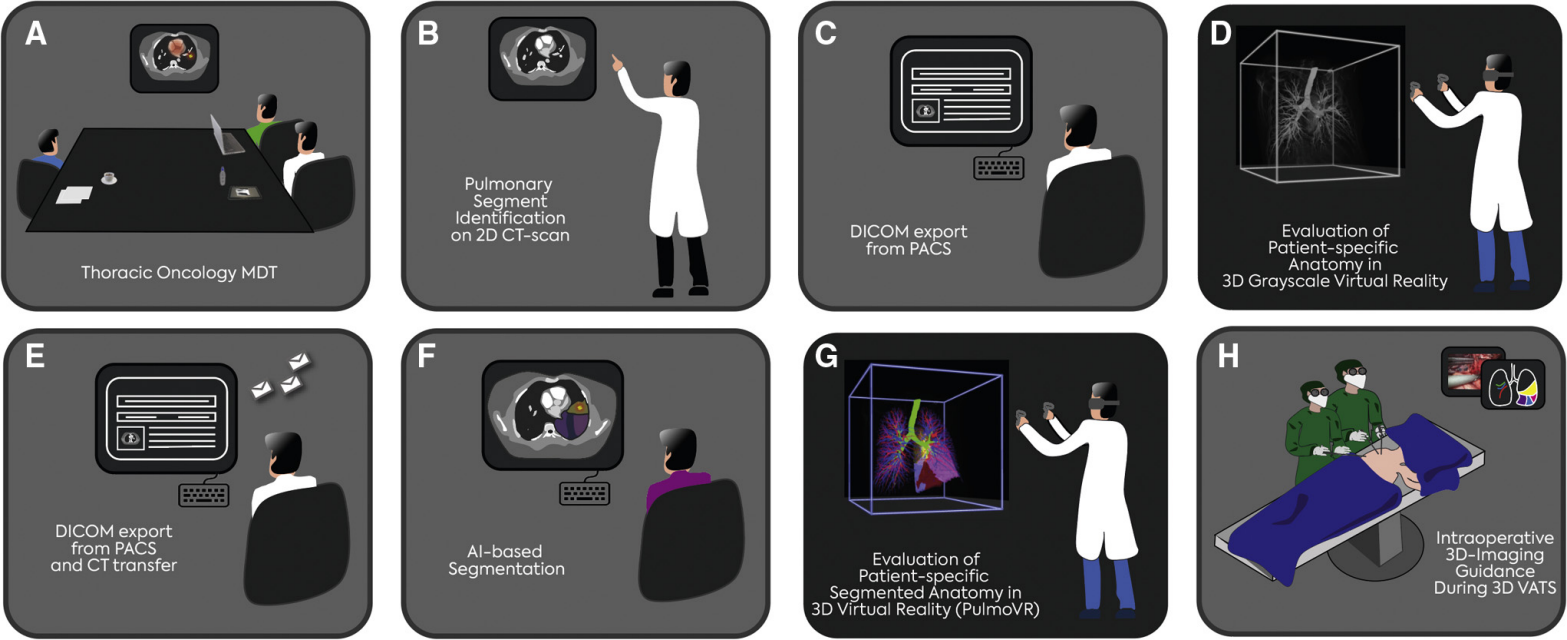
Schematic representation of virtual reality-based preoperative and intraoperative clinical workflow. A and B, After referral through our local thoracic oncology multidisciplinary team meeting a patient's anatomy is reviewed (by a surgeon) and the target segment is selected on conventional 2-dimensional-computed tomography (*2D-CT*) scan images. C and D, Digital imaging and communication in medicine (*DICOM*) files of CT scans are extracted from the local patient archiving and communication system (*PACS*) and reviewed in a 3-dimensional (*3D*) virtual reality (VR)-based DICOM viewer. E and F, Within this virtual environment, the surgeon can review grayscale patient-specific lung anatomy in 3D within a virtual reality digital room. After that, anonymized DICOM files are transferred for segmentation by an artificial intelligence (*AI*)-based algorithm. This algorithm automatically segments (ie, highlights) anatomic structures including arteries, veins, bronchi, and segments within a CT scan. G, Subsequently, DICOM files together with the segmentation files can be loaded into PulmoVR software (jointly developed and manufactured by Department of Cardiothoracic Surgery [Erasmus Medical Center], MedicalVR, EVOCS Medical Image Communication [Fysicon BV], and Thirona) on a VR workstation and 3D in-color VR-based images can be evaluated by the surgeon for preoperative planning of segmentectomy. H, During 3D video-assisted thoracoscopic surgery (VATS), static 3D reconstructions can be presented on monitors in the operating room to provide intraoperative guidance. *MDT*, Multidisciplinary team meeting.

The medical ethical committee of the Erasmus Medical Center approved this study (MEC-2020-0702). A total number of 10 consecutive patients who were eligible and accepted for pulmonary segmentectomy at the Erasmus Medical Center, Rotterdam, The Netherlands, were enrolled in this prospective study. All patients were consecutively included after referral and confirmation of eligibility for segmentectomy by the institutional local thoracic oncology multidisciplinary team meeting (Figure 1, *A*). After approval of a patient's eligibility for surgery, patients were planned for VATS segmentectomy. Subsequently, written informed consent was obtained from all patients and patients were included prospectively for VR-guided planning of segmentectomy as a supplement to conventional CT-guided planning. The inclusion criteria were as follows: age >18 years and pulmonary pathology suitable for pulmonary segmentectomy (ie, confirmed/clinical suspicion of stage I NSCLC ≤2 cm, limited benign lesion, and intrapulmonary metastases of other primary malignancies).

### Study Participants

Two of our dedicated lung surgeons and a surgery resident physician participated in the study. All participants received a brief (∼15 minutes) audiovisual tutorial on how to use the software and hardware before the start of the study.

### VR- and AI-based Surgical Planning Modalities

An immersive VR-based CT (grayscale) DICOM viewer was developed together with MedicalVR.^13^ In addition, a dedicated 3D-VR and AI-based pulmonary segmentectomy planning tool called PulmoVR was successfully developed together with MedicalVR, EVOCS Medical Image Communication (Fysicon BV), and Thirona. Segmental anatomy was determined automatically using LungQ software (Thirona). The AI-based delineation of pulmonary segments was based on determination of the bronchial and arterial intersegmental planes. Based on this delineation, the intersegmental veins were located within the border of segments. The anatomical 3D segmentations were visually checked by trained analysts for accuracy and corrected, as needed, within Thirona's ISO 13485-certified image analysis service. There were no specific technical requirements for 3D immersive evaluation of CT scans in VR besides a maximum of 1000 CT images and at least an adequate image quality for 2D evaluation. The AI analysis was possible on both contrast and noncontrast-enhanced inspiratory thoracic CT scans with a maximum slice thickness of 1.5 mm.

### Preoperative Planning Workflow

**Figure 2:**
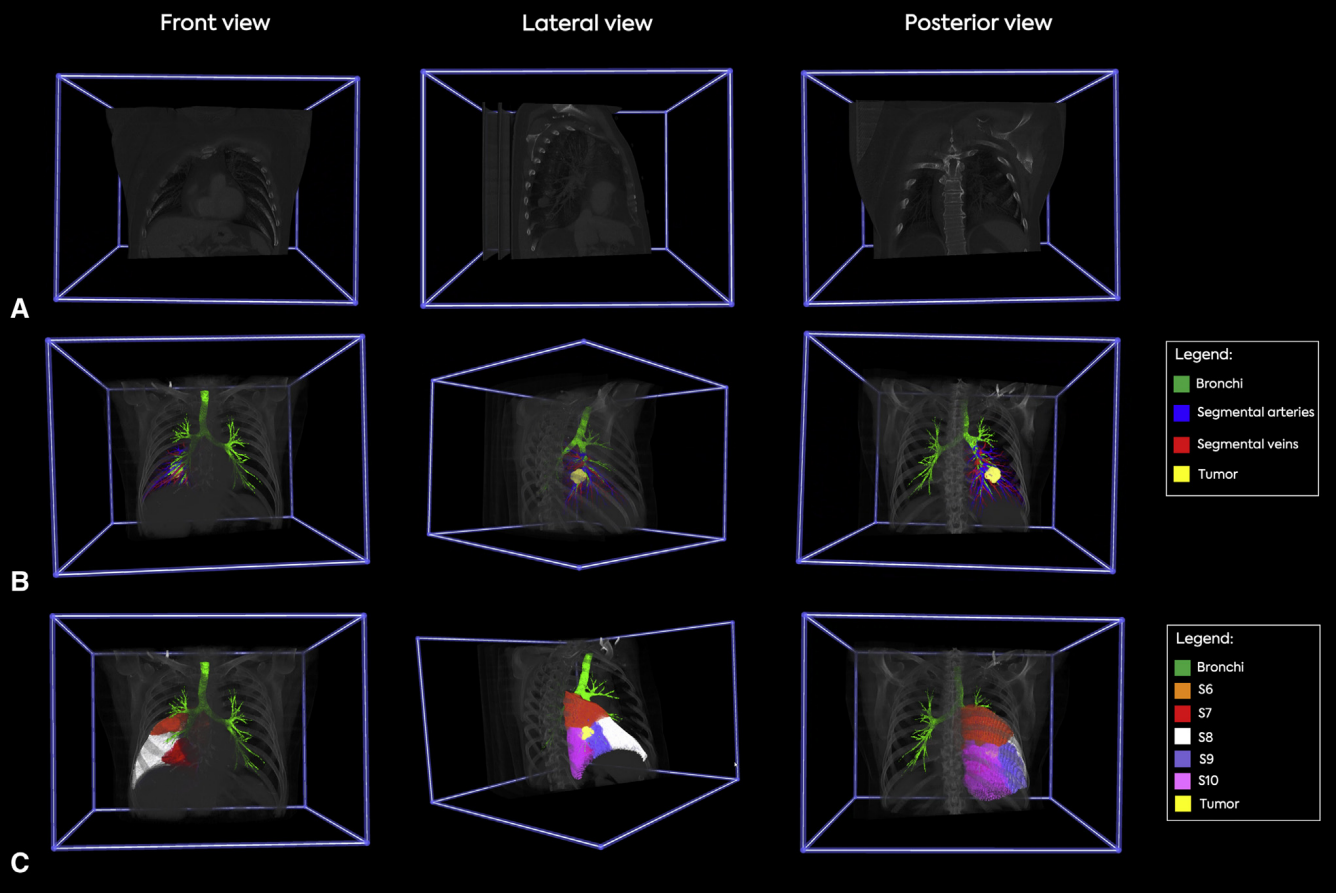
Evaluation of 3-dimensional (3D) reconstructions of pulmonary segmental anatomy in 3D virtual reality (VR) and PulmoVR (jointly developed and manufactured by Department of Cardiothoracic Surgery [Erasmus Medical Center], MedicalVR, EVOCS Medical Image Communication [Fysicon BV], and Thirona) for preoperative planning. A, A presentation of grayscale 3D-VR images from three different angles and cut planes. These images represent screenshots of the 3D models in virtual reality. B, PulmoVR views of a patient's segmental anatomy in which the tumor (*yellow*), bronchi (*green*), and segmental arteries (*blue*)/veins (*red*) of the right lower lobe are highlighted in different colors. C, PulmoVR-views of the same patient with segmentation of all right lower lobe anatomical segments (S); S6 (*orange*), S7 (*red*), S8 (*white*), S9 (*purple*), S10 (*pink*), bronchi (*green*), and tumor (*yellow*). Middle panel, Lateral view demonstrates the location of the tumor at the intersection of S6 (*orange*), S9 (*purple*), and S10 (*pink*). Note: Quality and in-depth perception are significantly higher when viewed in VR.

**Figure E1:**
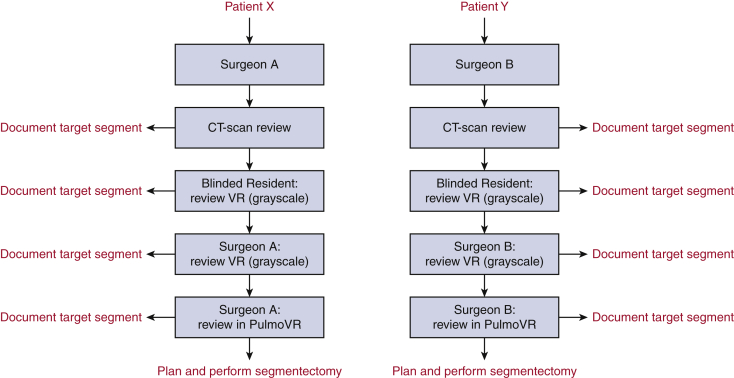
A schematic workflow on the preoperative workup carried out by the surgical team to prepare for segmentectomy. *CT*, Computed tomography; *VR*, virtual reality.

A schematic overview of the preoperative planning workflow is outlined in Figure 1 and Figure E1. After referral by our local multidisciplinary team (Figure 1, *A*), a surgeon independently identified and documented the target segment(s) on preoperative diagnostic CT scans. (Figure 1, *B*). Subsequently, the DICOM files were extracted from the patient archiving and communication system (Figure 1, *C*) and VR-based evaluation was carried out by a cardiothoracic surgery resident physician who was blinded to the study to independently evaluate the anatomy in our 3D-VR DICOM viewer (Figure 1, *D*). In this phase, the VR-based images were only a grayscale, 3D volume rendering of the CT scan without postprocessing edits (Figure 1, *D*, and Figure 2, *A*). The resident physician identified the lesion first, after which he identified the target segment based on anatomical landmarks (eg, intersegmental vein). Next, a surgeon also reviewed the anatomy in 3D-VR (Figure E1). The DICOM files were then transferred to Thirona and the pulmonary arteries, veins, segments, and the bronchi were segmented using LungQ (Thirona) AI-based lung quantification software (Figure 2, *F*). These analyses were transferred to our hospital within 72 hours. Then, PulmoVR enabled 3D visualization of both CT images and AI-based segmentation (segmentation means digital labeling of anatomic structures) files in immersive VR (Figure 2, *G*). Reconstructions were created for all patients undergoing segmentectomy and the target segment and patient-specific anatomy was evaluated in PulmoVR for the final time by the surgeon (Figure 2, *G*).

During surgery, a VR workstation was located in the operating theatre and provided an additional review of the anatomy when necessary. Moreover, preoperatively acquired 3D reconstructions of anatomy were displayed on operating theatre monitors intraoperatively. (Figure 2, *H*).

### Surgical Technique

Two dedicated cardiothoracic surgeons performed the 3D VATS segmentectomies independently (6 and 4 each). Surgical assistance was provided by a resident physician. All patients underwent 3D 3-port VATS under general anesthesia and double-lumen intubation and single lung ventilation were applied. Intraoperative intersegmental border demarcation was achieved using intravenous indocyanine green as described previously by Iizuka and colleagues.^14^ Transection of parenchyma was performed with endostaplers.

### Clinical End Point and Qualitative Assessment

All data were analyzed using Microsoft Office Excel 2015 (Microsoft, Redmond, Wash). This study aimed to study technical feasibility on the development of an AI- and VR-based tool for preoperative planning and performance of segmentectomy. Technical feasibility is defined as the ability to create an AI-based segmentation of patient-specific CT scans and to subsequently review pulmonary arteries, veins, bronchi, and segmental planes in an immersive VR application. The following clinical results were selected to serve as a clinical end point to demonstrate potential clinical safety: success of segmentectomy, resection margin (radical R0 resection), and intraoperative blood loss.

The participating cardiothoracic surgeons were asked to fill out a questionnaire (Appendix E1) after each procedure to assess intraoperative anatomy correspondence with their VR-based planning images. Additional questionnaires were filled out by the surgeons to evaluate the ease of use, satisfaction, and attitude toward future use of VR-guided surgical planning with PulmoVR (Appendix E2). These qualitative results served only as a subjective measurement to illustrate the potential usability of VR-based surgical planning.

## Results

### Patient Characteristics

A total of 10 consecutive patients (5 men and 5 women; mean age, 51 ± 13.4 years) were referred for elective segmentectomy and were enrolled in this study (Table 1). All baseline patient characteristics, pathology results, and target segments are outlined in Table 1.

**Table 1:** Clinical patient characteristics, surgical strategy, and perioperative outcome

Patient	Sex	Age (y)	Indication for segmentectomy	Target segment and surgical plan (after 2D-CT evaluation)	Adjusted target segment and surgical plan after 3D evaluation	Confirmation of target segment and surgical plan after review in PulmoVR∗	Final pathology	Resection margin (cm)	Tumor size (cm)	Intraoperative blood loss (mL)
1	Male	71	Metastasis (colorectal carcinoma)	RLL S9 (lateral basal segment)	RLL S9 + S10 + en-bloc wedge resection S6 (lateral, posterior and superior segment)	✓	Colorectal carcinoma metastasis	1.5 (R0)	5	100
2	Female	65	Primary NSCLC (adenocarcinoma)	RLL S6 (superior segment)	N/A	✓	Adenocarcinoma	1 (R0)	1.2	75
3	Female	64	Primary NSCLC (clinical suspicion)	LUL S1 (apical segment)	N/A	✓	Adenocarcinoma	1.7 (R0)	1	600
4	Male	56	Suspicious for metastasis (hypopharyngeal carcinoma)	RUL S1 (apical segment)	RUL S1 + en-bloc wedge resection RUL S2 (apical and posterior segment)	✓	Squamous cell carcinoma	1.7 (R0)	2.2	0
5	Male	55	Metastasis (para-ganglioma)	LLL S7/8 (medial basal segment)	N/A	✓	Para-ganglioma metastasis	0.4 (R0)	0.9	20
6	Female	41	Bronchiectasis	LUL S4+5 (superior and inferior lingular segment)	N/A	✓	Bronchiectasis with chronic infection	N/A	N/A	10
7	Male	31	Metastasis (yolk-sac tumor)	RLL S6 (superior segment)	RLL S8 (anterior segment)	✓	Metastasis germ-cell tumor (mature teratoma)	0.8 (R0)	0.8	5
8	Female	50	Primary NSCLC (clinical suspicion)	RLL S6 (superior segment)	N/A	✓	Adenocarcinoma	0.8 (R0)	1.4	10
9	Female	37	Suspicious for metastasis/loco-regional recurrence of stage IV NSCLC	LUL S4+5 (superior and inferior lingular segment)	LUL S5 (inferior lingular segment)	✓	Necrotizing granulomatous inflammation	N/A	N/A	0
10	Male	40	Suspicious for metastasis/loco-regional recurrence of stage IV NSCLC	LUL S1-3 (apical, posterior, and anterior segment)	N/A	✓	Adenocarcinoma	2.5 (R0)	2.8	200

*2D-CT*, 2-Dimensional computed tomography; *3D*, 3-dimensional; *RLL*, right lower lobe; *NSCLS*, non–small cell lung cancer; *N/A*, not available; *LUL,* left upper lobe; *RUL,* right upper lobe; *LLL*, left lower lobe.

∗ PulmoVR was jointly developed and manufactured by Department of Cardiothoracic Surgery [Erasmus Medical Center], MedicalVR, EVOCS Medical Image Communication (Fysicon BV), and Thirona.

### Technical Feasibility: 3D-VR and PulmoVR Reconstructions

**Figure 3:**
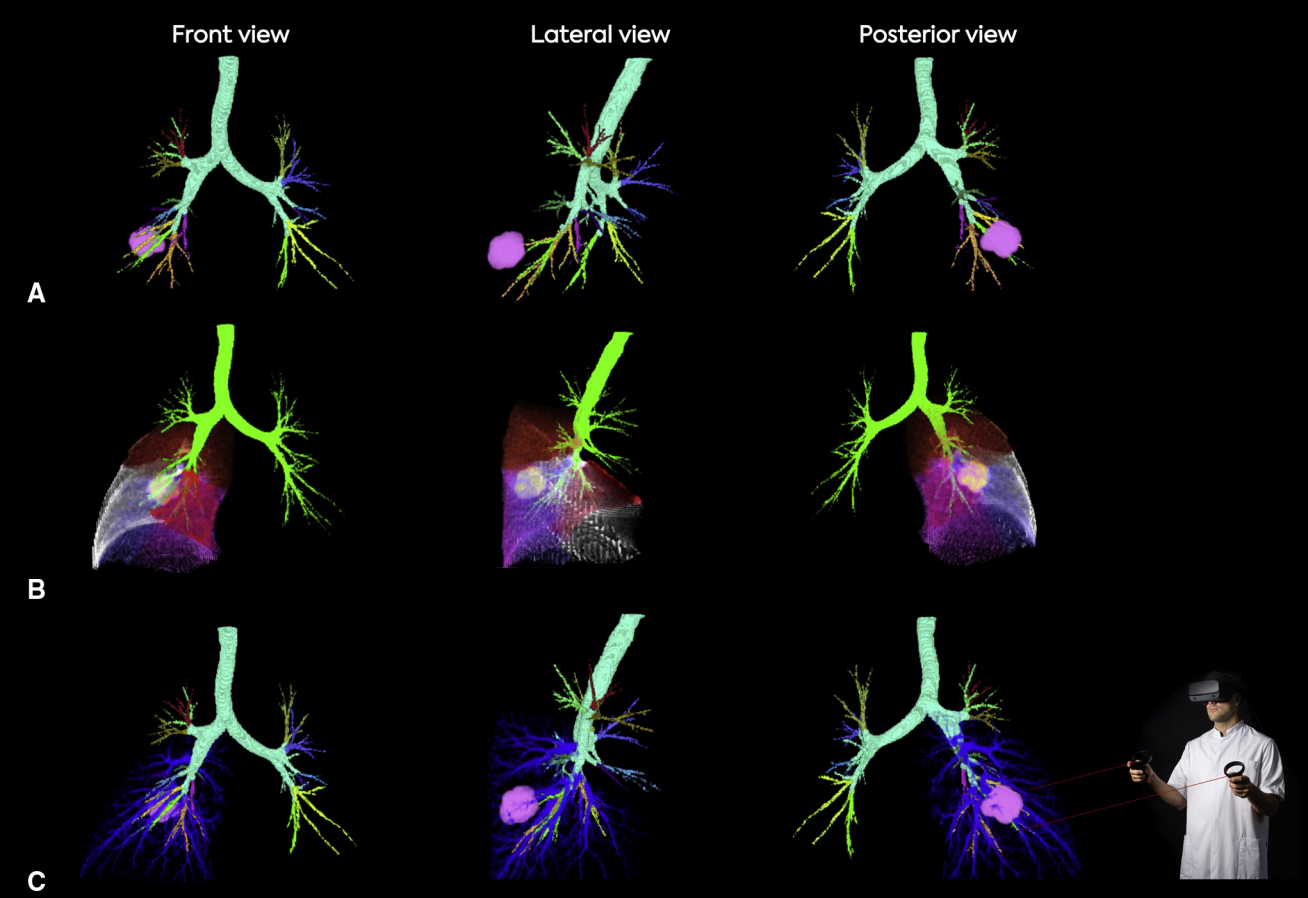
Selective segmentation and visualization of patient-specific anatomy in PulmoVR (jointly developed and manufactured by Department of Cardiothoracic Surgery [Erasmus Medical Center], MedicalVR, EVOCS Medical Image Communication [Fysicon BV], and Thirona). A, All segmental bronchi and the tumor (*pink*) are highlighted in different colors (*top and bottom line*) and shown from 3 different angles (*front*, *lateral*, and *posterior*). B, The pulmonary segments are translucent and the tumor (*yellow in middle and pink in top and bottom line*) extension through segmental borders can be seen when viewed from different angles. C, Pulmonary artery segmental branches are highlighted in blue to evaluate the relation of segmental arteries with the tumor (*pink*). Note: Quality and in-depth perception are much higher when viewed in virtual reality.

In all patients, VR reconstructions were made successfully. As demonstrated in Figures 2 and 3, for all patients, the segmental arteries, segmental veins, bronchi, and segmental borders were visualized in PulmoVR. Segmentations of vasculature and segmental borders were made only in the lobe of interest (Figures 2 and 3). All other thoracic structures were automatically visualized in grayscale as well. Based on the questionnaire that was filled out by the surgeons postoperatively after each case (Appendix E1), in 100% of the cases, intraoperative anatomy corresponded entirely with preoperative VR images. Videos 1 and 2 demonstrate the VR-guided evaluation of the intrapulmonary segmental anatomy from various perspectives in 2 different cases.

### Clinical Applicability: Perioperative Outcomes and Surgical Adjustments

All segmentectomy procedures were performed successfully through 3D VATS, assisted by preoperative VR planning. A complete resection (ie, R0) with adequate surgical margins was obtained in all cases (Table 1).

In 4 out of 10 cases, the VR-based evaluation resulted in a change in the selection of the target segment than the initially selected target segment by 2D-CT. We will briefly highlight these 4 cases. For all operations, initial surgical strategies and changes after review in VR are outlined in Table 1.

#### Patient 1

A patient with colorectal metastasis in the right lower lobe was referred for segmentectomy of segment (S) 9. In this patient VR evaluation showed tumor expansion through the segmental borders of S6, S9, and S10. Consequently, surgical strategy after evaluation in VR was adjusted accordingly and en-bloc segmentectomy of S9 and S10 and a subsegmentectomy of S6 was performed (see Figures 2 and 3 for VR images of this patient).

#### Patient 4

**Figure 4:**
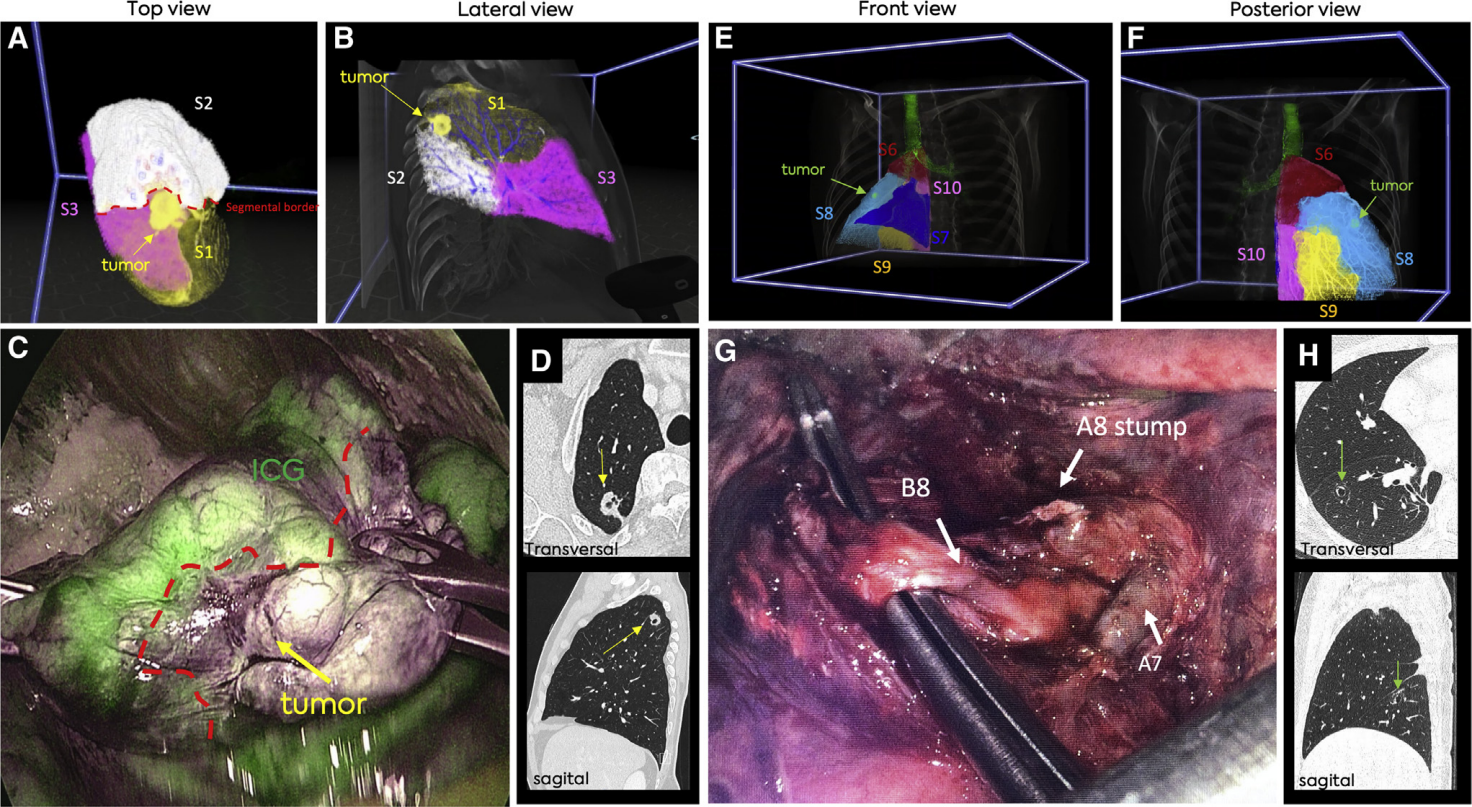
Two representative cases of virtual reality (VR)-adjusted segmentectomy planning. A and B, Two snapshots of the VR-rendered images in a patient with a tumor in segment (*S*) 1. The tumor (*yellow arrow*) and its small margins to S2 are visualized (*red dotted line*). C, Intraoperative screenshot of video-assisted thoracoscopic surgery (VATS) during indocyanine green (*ICG*)-based demarcation of the segmental border (*red dotted line*) between S1 and S2, showing correspondence with the VR images in A and B. D, Representative snapshots of transversal and sagittal computed tomography scan of patient 4. E and F, Two snapshots of patient 7 with a tumor (*green arrow*) in S8. The close relation of the tumor with the lower part of S6 can be seen in panel F. G, Intraoperative screenshot of VATS after ligation of segmental artery (A) 8. H, Representative snapshots of transversal and sagittal computed tomography scan of patient 7. *B8*, Segmental bronchus 8.

A patient with a tumor in the apico-dorsal part of S1 of the right upper lobe was referred for segmentectomy of S1. VR evaluation showed the relatively small tumor margin in relation with the S1/S2 border (Figure 4, *A* and *B*), which was not visible on preoperative CT (Figure 4, *D*). Consequently, the patient underwent an en-bloc resection of S1 and a subsegment of S2 to maintain safe surgical margins. Intraoperatively obtained indocyanine green-guided intersegmental border visualization corresponded with the preoperatively acquired VR reconstructions (Figure 4, *C*).

#### Patient 7

Patient 7 had a history of pulmonary metastasized mixed germ cell tumor for which he underwent wedge resection of the right lower lobe during September 2019. During follow-up, there was a new pulmonary lesion (suspicious recurrent metastasis) for which he received chemotherapy. Chemotherapy did not fully resolve the lesion and the patient was accepted for redo VATS segmentectomy for the cystic lesion. After review in CT, the lesion was believed to be located in S6 (Figure 4, *H*). However, after evaluation in VR, the tumor seemed to be located clearly in S8 (Figure 4, *E* and *F*). A successful segmentectomy of S8 was performed (Figure 4, *G*).

#### Patient 9

A patient with a tumor in the lingula (suspicious for metastasis/loco-regional recurrence of stage IV NSCLC) based on CT images was referred for lingulectomy (S4 and S5). However, VR evaluation showed that the lesion was located entirely within S5 and consequently resulted in a successful segmentectomy of S5.

### Surgeons’ Experiences

Table 2 depicts the experience of the surgeons. Overall, PulmoVR was rated to be easy to use, useful, and efficient for preoperative planning of segmentectomy.

**Table 2:** Questionnaire results on the virtual reality experience of the participating surgeons

Question∗	Surgeon #1	Surgeon #2
Ease of use		
1. Learning to operate with PulmoVR† was easy	5	4
2. Rotating the CT scan in PulmoVR was easy	5	4
3. Cutting (slicing in 3D) through the CT scan was easy	5	4
4. Reviewing the CT scan from 360° was easy	5	4
5. Selectively reviewing the segmental anatomy (eg, arteries, veins, bronchi, and segmental borders) was easy	4	4
Usefulness and efficiency of PulmoVR		
1. PulmoVR helps me to review segmental anatomy more accurately compared to reviewing CT scans in 2D	5	5
2. The assessment pulmonary segmental anatomy in PulmoVR takes less time than conventional CT scan review	4	5
3. Segmentation (highlighting arteries, veins, and bronchi in color) of the CT scans in PulmoVR is of added value for CT assessment	5	5
4. PulmoVR helps me to prepare a surgical strategy more efficiently compared with conventional methods (eg, CT scans).	5	5
5. PulmoVR review of CT scans creates more awareness of spatial orientation of anatomical structures and therefore contributes to the safer performance of segmentectomies	5	5
Attitude toward future use of PulmoVR in preoperative planning of segmentectomy		
1. I would like to work with PulmoVR in the future	5	5
2. The future, I prefer using PulmoVR for preoperative planning as a supplement to conventional methods	5	5
3. In the future, I prefer using PulmoVR for preoperative planning instead of conventional methods	2	1
4. I believe, in the future, PulmoVR-like technologies will become a standard of care imaging modality for preoperative planning of segmentectomies	5	5
5. I would recommend PulmoVR as a preoperative planning tool to my colleagues	5	5

*CT*, Computed tomography; *3D*, 3 dimensional; *2D*, 2 dimensional.

∗ Scale was 1 = strongly disagree to 5 = strongly agree.

† PulmoVR was jointly developed and manufactured by Department of Cardiothoracic Surgery, MedicalVR, EVOCS Medical Image Communication (Fysicon BV), and Thirona.

## Discussion

In general, pulmonary segmentectomies are considered technically challenging and difficult procedures, especially when compared with lobectomy or other sublobar resections, such as wedge resection. Several factors have been identified that contribute to the technical difficulties of segmentectomy, including understanding of the individual pulmonary anatomy and its relation to the tumor localization. Accurate evaluation of segmental pulmonary anatomy remains a challenge when assessed on 2D-CT images. For this reason, we codeveloped an innovative AI and immersive VR-based segmentectomy planning tool (the PulmoVR). In this study we report our early results on the technical feasibility and clinical applicability of our PulmoVR application in patients undergoing VATS segmentectomy.

Recent studies have explored and highlighted the role of 3D-CT angiography and manual 3D virtual modeling of CT images to examine patient-specific (segmental) anatomy preoperatively.^15^, ^16^, ^17^ Moreover, reports from other studies that have used custom-made 3D software to simulate a segmentectomy procedure have been published and underline the benefits that 3D modeling offers in the context of segmentectomy planning.^15^^,^^17^

An important disadvantage of 3D modeling is the loss of data because most often these applications focus on the visualization of specifically selected and requested anatomic structures such as pulmonary vessels, tumor, bronchi, and parenchyma.^16^ As a result, information on intrapulmonary lymph nodes, thoracic cage anatomy (eg, intercostal spaces and sternum), the heart, diaphragm, and other intrathoracic vessels is lost or needs to be selected and manually segmented separately. Our 3D-VR DICOM viewer, as described in the current study, enables direct VR visualization by virtually stacking the CT images and allowing evaluation in VR. This provides a fast method (<1 minute) to evaluate patient-specific CT scans in 3D and with reality-like in-depth perception in 360° without the need for postprocessing techniques and without loss of imaging data.^11^^,^^12^

Furthermore, in currently available techniques 3D segmentation (which is the identification and selective coloring of an anatomic structure) is often performed manually (by technicians), which increases the risk for error, requires manual labor, is time-consuming, and for this reason is more expensive.^18^ Another shortcoming of the available 3D modeling software is that it commonly requires contrast-enhanced CT scanning for accurate segmentation, which results in radiation and iodine-contrast agent exposure and consequently in additional health risks for patients.^18^ In this study, we have demonstrated that our PulmoVR application provides a unique environment to review patient-specific segmental anatomy by facilitating selective AI-based automatic labeling, visualization, and assessment of pulmonary structures, without the need for contrast-enhanced CT imaging. Moreover, automated imaging algorithms could potentially create more efficient planning.

In addition, 3D imaging review on a computer screen remains a 3D visualization on a 2D monitor and requires extensive (and separate) review of pulmonary structures from different angles to understand the spatial relations and in-depth anatomy of structures. Finally, obtaining accurate 3D images in this case also requires additional investment in hardware (3D monitors, high-performance computers, and glasses). In accordance to the reported data from the literature on VR applications, in this study we have demonstrated that our PulmoVR tool enables immersive interactive image manipulation, realistic in-depth perception, and immersive visualization of complex relationships of anatomic structures (eg, vascular, bronchial, tumor margins, and segmental borders) that can be applied by surgeons preoperatively (without the need to consult radiologists or imaging technicians) to gain a better and more realistic insight into a patient's specific anatomy.^8^^,^^10^ Additionally, we have showed that PulmoVR tool is easy and ready to use after only a single demonstration and tutorial of 15 minutes as was concluded from the questioners filled out by the participating surgeons. The software can be installed on every computer or laptop with sufficient graphical and processing power and the off-the-shelf VR head mounted displays are widely available. There is no need for additional 3D screens, hardware, or glasses.

### Future Outlook

In more comparative research we aim to study the application of PulmoVR in larger patient populations. In addition, our goal is to design a multiuser platform in which a patient's anatomy can be evaluated by more users at the same time. Hopefully, this would also pave the way for the development of surgical simulations for education and training purposes.

Other exciting and futuristic VR applications in surgical planning might be the application of VR or augmented reality to enhance intraoperative views in robotic procedures, such as robotic segmentectomies. In terms of preoperative planning, VR can assess the ideal thoracoscopic port placement locations. Also, the intraoperative 3D view could be merged with preoperatively acquired reconstructions. This could potentially enhance intraoperative navigation and also the identification of very small intrapulmonary lesions, lymph nodes, and small vascular branches. Other interesting examples of VR-based guidance and planning are navigational (ie, VR guided) bronchoscopy or augmented reality guided detection of small and deeply located lesions.^9^^,^^19^ Further development and use of these novel technologies could potentially result in better preoperative diagnostics.

### Limitations

We acknowledge that the current pilot study has some limitations in terms of technology and study setup. First, only a small number of patients were included, and only 2 cardiothoracic surgeons have used and evaluated the platform. To draw definite conclusions to structurally implement such technology, more prospective, extensive, and comparative analyses are required. Also, cost-effectiveness studies are important to evaluate implementation in the clinic. In addition, in the current situation, only segmental branches of blood vessels are visualized in PulmoVR. To further improve on the software, the entire pulmonary artery should ideally be segmented to ease evaluation in PulmoVR. Ideally, also (semi)automatic segmentation of intrapulmonary and mediastinal lymph nodes could ease surgical planning and performance of lymph node dissection.

Another important limitation of PulmoVR is the lack of the possibility to simulate lung deflation, manipulation, and virtual resection/transection. As a result, PulmoVR cannot yet be used to create augmented virtual projections of anatomy within the physical surgical field. Future development of augmented reality and digital navigation systems should hopefully overcome this challenge within the next decade.

## Conclusions

**Figure 5:**
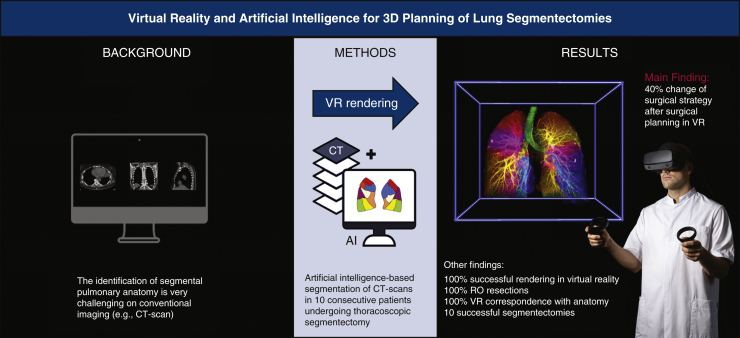
The identification of segmental anatomy for surgical planning of lung segmentectomy is quite challenging with conventional imaging. In the current study, a novel method is presented to create artificial intelligence based virtual reality reconstructions to prepare for thoracoscopic segmentectomies in 10 consecutive patients. *CT*, Computed tomography; *VR,* virtual reality.

This study presents the first artificial intelligence and immersive VR-based visualization method for segmentectomy planning (Figure 5). We have demonstrated technical feasibility and clinical applicability in 10 consecutive patients undergoing thoracoscopic segmentectomy. Notably, the application of PulmoVR resulted in essential changes in surgical strategy in 40% of the cases. All segmentectomy procedures were performed successfully, and all oncologic resections adequately. More extensive clinical trials are needed to study safety and clinical benefits and to provide clinical evidence for the application of virtual reality in segmentectomy planning.

### Conflict of Interest Statement

Drs Sadeghi and Mahtab are coinventors of the virtual reality-based technology presented in this article. All other authors reported no conflicts of interest. This work was supported by Koers23, Erasmus Medical Center, The Netherlands.

The *Journal* policy requires editors and reviewers to disclose conflicts of interest and to decline handling or reviewing manuscripts for which they may have a conflict of interest. The editors and reviewers of this article have no conflicts of interest.

## Figures and Tables

**Table:** 

**5** **Strongly Agree**	**4** **Agree**	**3** **Neither Or N/A**	**2** **Disagree**	**1** **Strongly Disagree**

**Table:** 

**5** **Strongly Agree**	**4** **Agree**	**3** **Neither Or N/A**	**2** **Disagree**	**1** **Strongly Disagree**

**Table:** 

**5** **Strongly Agree**	**4** **Agree**	**3** **Neither Or N/A**	**2** **Disagree**	**1** **Strongly Disagree**

**Table:** 

**5** **Strongly Agree**	**4** **Agree**	**3** **Neither Or N/A**	**2** **Disagree**	**1** **Strongly Disagree**

**Table:** 

**5** **Strongly Agree**	**4** **Agree**	**3** **Neither Or N/A**	**2** **Disagree**	**1** **Strongly Disagree**

**Table:** 

**5** **Strongly Agree**	**4** **Agree**	**3** **Neither Or N/A**	**2** **Disagree**	**1** **Strongly Disagree**

**Table:** 

**5** **Strongly Agree**	**4** **Agree**	**3** **Neither Or N/A**	**2** **Disagree**	**1** **Strongly Disagree**

**Table:** 

**5** **Strongly Agree**	**4** **Agree**	**3** **Neither Or N/A**	**2** **Disagree**	**1** **Strongly Disagree**

**Table:** 

**5** **Strongly Agree**	**4** **Agree**	**3** **Neither Or N/A**	**2** **Disagree**	**1** **Strongly Disagree**

**Table:** 

**5** **Strongly Agree**	**4** **Agree**	**3** **Neither Or N/A**	**2** **Disagree**	**1** **Strongly Disagree**

**Table:** 

**5** **Strongly Agree**	**4** **Agree**	**3** **Neither Or N/A**	**2** **Disagree**	**1** **Strongly Disagree**

**Table:** 

**5** **Strongly Agree**	**4** **Agree**	**3** **Neither Or N/A**	**2** **Disagree**	**1** **Strongly Disagree**

**Table:** 

**5** **Strongly Agree**	**4** **Agree**	**3** **Neither Or N/A**	**2** **Disagree**	**1** **Strongly Disagree**

**Table:** 

**5** **Strongly Agree**	**4** **Agree**	**3** **Neither Or N/A**	**2** **Disagree**	**1** **Strongly Disagree**

**Table:** 

**5** **Strongly Agree**	**4** **Agree**	**3** **Neither Or N/A**	**2** **Disagree**	**1** **Strongly Disagree**

## Appendix E1

Questionnaire presented to surgeons after each procedure to assess intraoperative anatomy correspondence with their virtual reality-based planning images. PulmoVR was jointly developed and manufactured by Department of Cardiothoracic Surgery (Erasmus Medical Center), MedicalVR, EVOCS Medical Image Communication (Fysicon BV), and Thirona.

Please answer the following questions regarding correspondence of virtual reality-guided planning images and intraoperative anatomy findings:

**Pulmonary segmental arteries: Intraoperative findings corresponded with the preoperative PulmoVR images**

Agree/Disagree

**Pulmonary segmental veins: Intraoperative findings corresponded with the preoperative PulmoVR images**

Agree/Disagree

**Pulmonary segmental bronchial anatomy: Intraoperative findings corresponded with the preoperative PulmoVR images**

Agree/Disagree

## Appendix E2

Questionnaire filled out by the surgeons to evaluate the ease of use, satisfaction, and attitude toward future use of virtual reality-guided surgical planning with PulmoVR (jointly developed and manufactured by Department of Cardiothoracic Surgery [Erasmus Medical Center], MedicalVR, EVOCS Medical Image Communication [Fysicon BV], and Thirona). *CT*, Computed tomography; *N/A*, not applicable; *3D*, 3 dimensional.

### Ease of Use

1. Learning to operate with PulmoVR was easy.

2. Rotating the CT scan in PulmoVR was easy.

3. Cutting (slicing in 3D) through the CT scan was easy.

4. Reviewing the CT scan from 360° was easy.

5. Selectively reviewing the segmental anatomy (eg, arteries, veins, bronchi, and segmental borders) was easy.

### Usefulness and Efficiency of PulmoVR

1. PulmoVR helps me to review segmental anatomy more accurately compared to reviewing CT scans in 2D.

2. The assessment pulmonary segmental anatomy in PulmoVR takes less time than conventional CT-scan review.

3. Segmentation (highlighting arteries, veins, bronchi in color) of the CT scans in PulmoVR is of added value for CT assessment.

4. PulmoVR helps me to prepare a surgical strategy more efficiently compared to conventional methods (eg CT-scans)

5. PulmoVR review of CT scans creates more awareness of spatial orientation of anatomical structures and therefore contributes to the safer performance of segmentectomies.

### Attitude Towards Future Use of PulmoVR in Planning Segmentectomy

1. I would like to work with PulmoVR in the future.

2. In the future, I prefer using PulmoVR for preoperative planning **as a supplement** to conventional methods.

3. In the future, I prefer using PulmoVR for preoperative planning **instead** of conventional methods.

4. I believe, in the future, PulmoVR-like technologies will become a standard of care imaging modality for preoperative planning of segmentectomies

5. I would recommend PulmoVR as a preoperative planning tool to my colleagues

## Supplementary Data

Video 1 Virtual reality (VR)-based view of a patient-specific scan with a tumor in the right lower lobe. The review of the anatomy is performed by using VR controllers (Oculus Rift S [Oculus VR, Irvine, Calif]) that enable 360° rotation and zoom. Different segments of the right lower lobe are shown transparent in various colors. The bronchial anatomy (*green*) is also shown. Additionally, measurements can be performed in virtual reality to determine tumor size and expected surgical margins. Video available at: https://www.jtcvs.org/article/S2666-2507(21)00253-4/fulltext.

Video 2 Virtual reality (VR) representation of a patient's scan with a tumor in the right upper lobe. Segmental arteries (*blue*), veins (*red*), and bronchial anatomy (*green*) are shown. The evaluation of computed tomography scans can be performed on a computer monitor in 3 dimensions as well as in VR with VR controllers. In addition. our PulmoVR software (jointly developed and manufactured by Department of Cardiothoracic Surgery [Erasmus Medical Center], MedicalVR, EVOCS Medical Image Communication [Fysicon BV], and Thirona) enables to take notes during review of the scan to determine the segmental branches of the arterial supply and venous drainage. Video available at: https://www.jtcvs.org/article/S2666-2507(21)00253-4/fulltext.

## References

[1] Yang C.F., D'Amico T.A. (2012). Thoracoscopic segmentectomy for lung cancer. Ann Thorac Surg.

[2] Ghaly G., Kamel M., Nasar A., Paul S., Lee P.C., Port J.L. (2016). Video-assisted thoracoscopic surgery is a safe and effective alternative to thoracotomy for anatomical segmentectomy in patients with clinical stage I non–small cell lung cancer. Ann Thorac Surg.

[3] Marchand P., Gilroy J.C., Wilson V.H. (1950). An anatomical study of the bronchial vascular system and its variations in disease. Thorax.

[4] Cory R.A., Valentine E.J. (1959). Varying patterns of the lobar branches of the pulmonary artery. A study of 524 lungs and lobes seen at operation of 426 patients. Thorax.

[5] Oizumi H., Kanauchi N., Kato H., Endoh M., Suzuki J., Fukaya K. (2011). Anatomic thoracoscopic pulmonary segmentectomy under 3-dimensional multidetector computed tomography simulation: a report of 52 consecutive cases. J Thorac Cardiovasc Surg.

[6] Sardari Nia P., Olsthoorn J.R., Heuts S., Maessen J.G. (2019). Interactive 3D reconstruction of pulmonary anatomy for preoperative planning, virtual simulation, and intraoperative guiding in video-assisted thoracoscopic lung surgery. Innovations.

[7] Sadeghi A.H., Taverne Y., Bogers A., Mahtab E.A.F. (2020). Immersive virtual reality surgical planning of minimally invasive coronary artery bypass for Kawasaki disease. Eur Heart J.

[8] Shirk J.D., Thiel D.D., Wallen E.M., Linehan J.M., White W.M., Badani K.K. (2019). Effect of 3-dimensional virtual reality models for surgical planning of robotic-assisted partial nephrectomy on surgical outcomes: a randomized clinical trial. JAMA Netw Open.

[9] Maat A., Sadeghi A.H., Bogers A., Mahtab E. The realm of oncological lung surgery: from past to present and future perspectives. https://www.intechopen.com/books/update-in-respiratory-diseases/the-realm-of-oncological-lung-surgery-from-past-to-present-and-future-perspectives.

[10] Sadeghi A., Mathari S., Abjigitova D., Maat A.P.W.M., Taverne Y.J.H.J., Bogers A.J.J.C. (December 18, 2020). Current and future applications of virtual, augmented, and mixed reality in cardiothoracic surgery. Ann Thorac Surg.

[11] Lassen-Schmidt B.C., Kuhnigk J.M., Konrad O., van Ginneken B., van Rikxoort E.M. (2017). Fast interactive segmentation of the pulmonary lobes from thoracic computed tomography data. Phys Med Biol.

[12] Fischer A.M., Varga-Szemes A., Martin S.S., Sperl J.I., Sahbaee P., Neumann D. (2020). Artificial intelligence-based fully automated per lobe segmentation and emphysema-quantification based on chest computed tomography compared with global initiative for chronic obstructive lung disease severity of smokers. J Thorac Imaging.

[13] Sadeghi A.H., Bakhuis W., Van Schaagen F., Oei F.B., Bekkers J.A., Maat A.P. (2020). Immersive 3D virtual reality imaging in planning minimally invasive and complex adult cardiac surgery. Eur Heart J Digital Health.

[14] Iizuka S., Kuroda H., Yoshimura K., Dejima H., Seto K., Naomi A. (2016). Predictors of indocyanine green visualization during fluorescence imaging for segmental plane formation in thoracoscopic anatomical segmentectomy. J Thorac Dis.

[15] Yang Q., Xie B., Hu M., Sun X., Huang X., Guo M. (2016). Thoracoscopic anatomic pulmonary segmentectomy: a 3-dimensional guided imaging system for lung operations. Interact Cardiovasc Thorac Surg.

[16] Seguin-Givelet A., Grigoroiu M., Brian E., Gossot D. (2019). Planning and marking for thoracoscopic anatomical segmentectomies. J Thorac Dis.

[17] Saji H., Inoue T., Kato Y., Shimada Y., Hagiwara M., Kudo Y. (2013). Virtual segmentectomy based on high-quality three-dimensional lung modelling from computed tomography. Interact Cardiovasc Thorac Surg.

[18] Le Moal J., Peillon C., Dacher J.N., Baste J.M. (2018). Three-dimensional computed tomography reconstruction for operative planning in robotic segmentectomy: a pilot study. J Thorac Dis.

[19] Rouze S., de Latour B., Flecher E., Guihaire J., Castro M., Corre R. (2016). Small pulmonary nodule localization with cone beam computed tomography during video-assisted thoracic surgery: a feasibility study. Interact Cardiovasc Thorac Surg.

