# Supplementary figures and images for: Virtual reality and artificial intelligence for 3-dimensional planning of lung segmentectomies
Source: JTCVS Tech. 2021 Mar 16;7:309–21. doi: 10.1016/j.xjtc.2021.03.016 (PMC8312141; doi:10.1016/j.xjtc.2021.03.016)

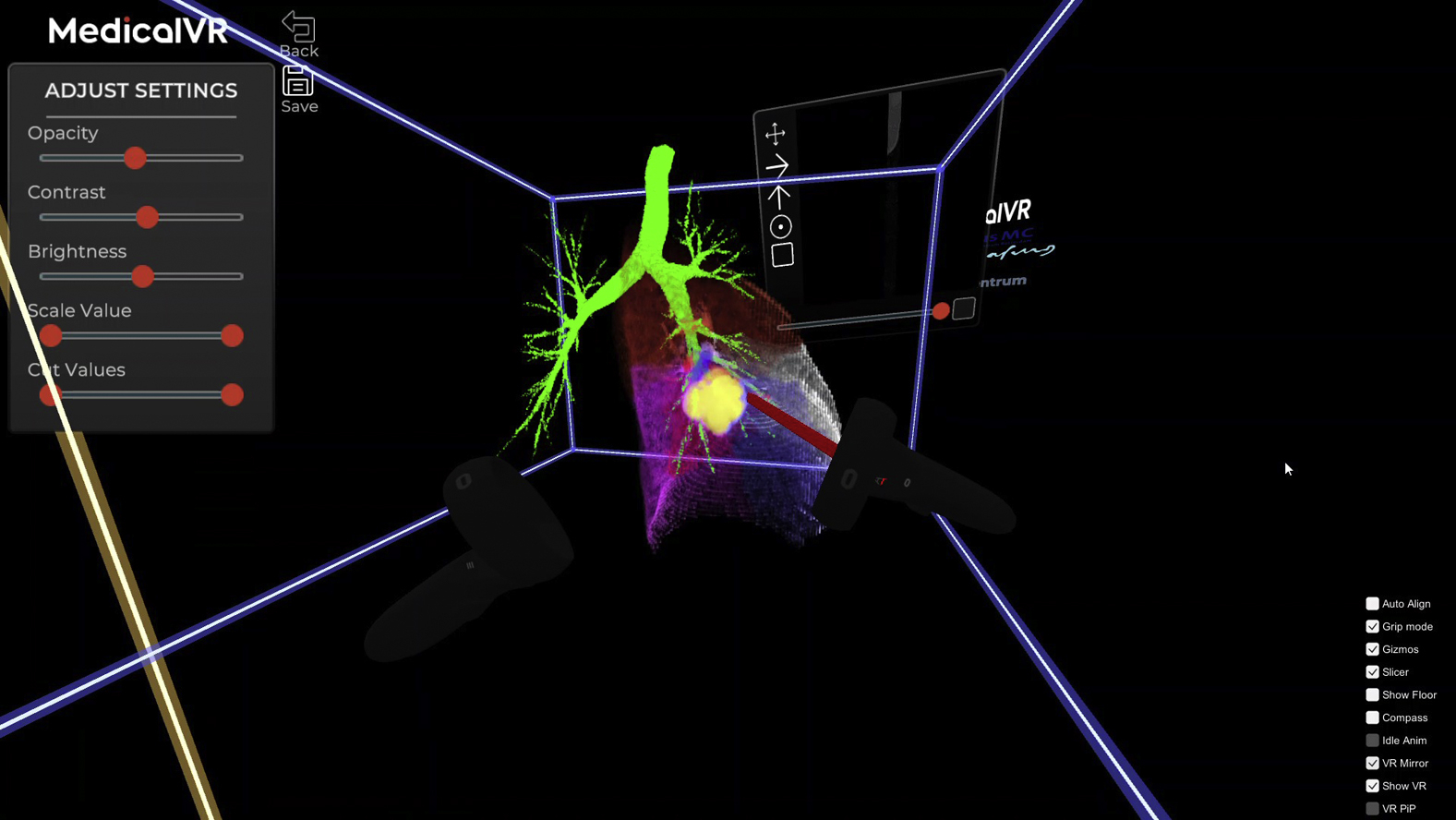

Supplement: Video 1 — Virtual reality (VR)-based view of a patient-specific scan with a tumor in the right lower lobe. The review of the anatomy is performed by using VR controllers (Oculus Rift S [Oculus VR, Irvine, Calif]) that enable 360° rotation and zoom. Different segments of the right lower lobe are shown transparent in various colors. The bronchial anatomy (green) is also shown. Additionally, measurements can be performed in virtual reality to determine tumor size and expected surgical margins. Video available at: https://www.jtcvs.org/article/S2666-2507(21)00253-4/fulltext. [file fx3.jpg]

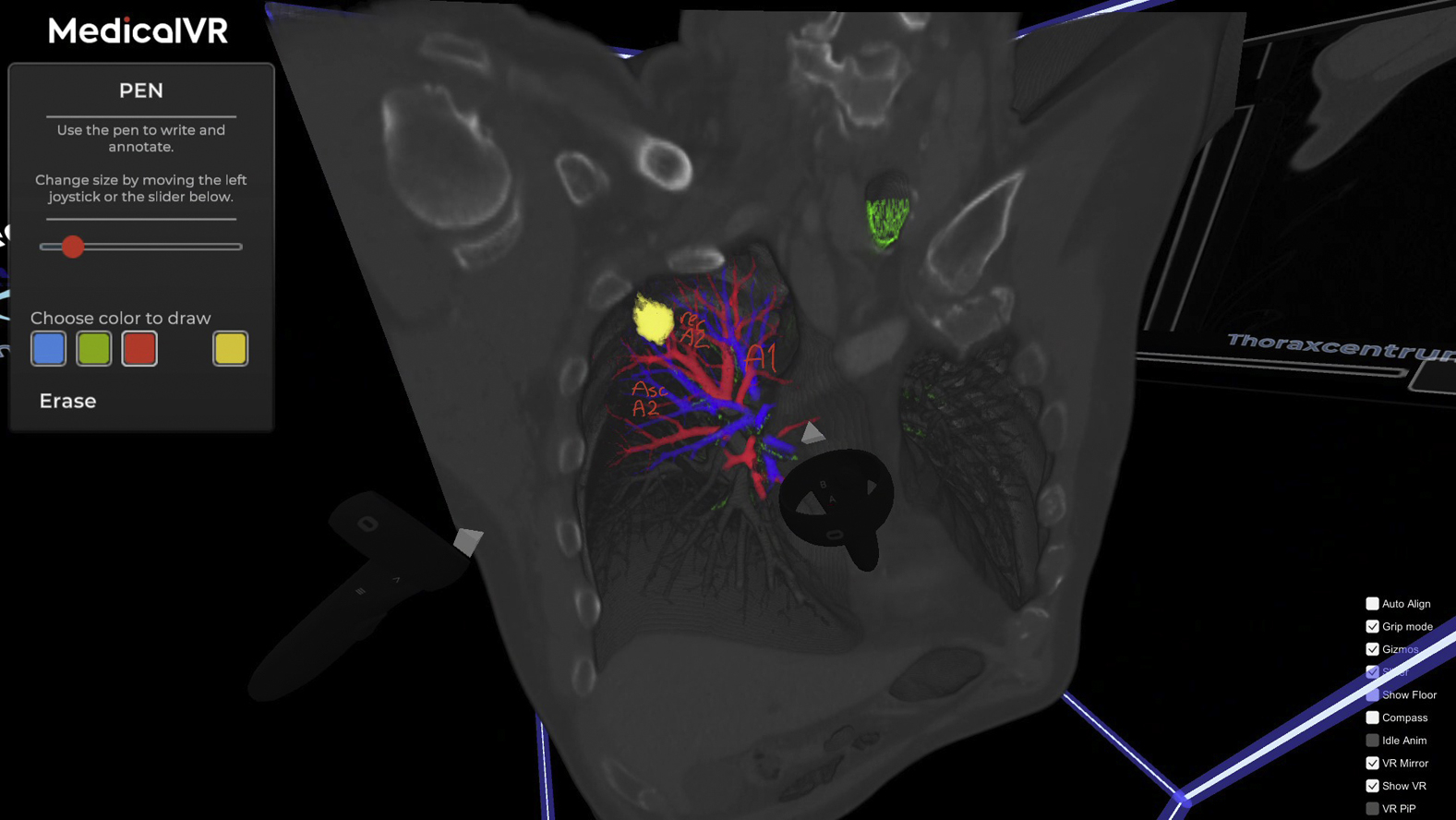

Supplement: Video 2 — Virtual reality (VR) representation of a patient's scan with a tumor in the right upper lobe. Segmental arteries (blue), veins (red), and bronchial anatomy (green) are shown. The evaluation of computed tomography scans can be performed on a computer monitor in 3 dimensions as well as in VR with VR controllers. In addition. our PulmoVR software (jointly developed and manufactured by Department of Cardiothoracic Surgery [Erasmus Medical Center], MedicalVR, EVOCS Medical Image Communication [Fysicon BV], and Thirona) enables to take notes during review of the scan to determine the segmental branches of the arterial supply and venous drainage. Video available at: https://www.jtcvs.org/article/S2666-2507(21)00253-4/fulltext. [file fx4.jpg]
